# All-CLL: A Capture-based Next-generation Sequencing Panel for the Molecular Characterization of Chronic Lymphocytic Leukemia

**DOI:** 10.1097/HS9.0000000000000962

**Published:** 2023-09-21

**Authors:** Irene López-Oreja, Mónica López-Guerra, Juan Correa, Pablo Mozas, Ana Muntañola, Luz Muñoz, Ana-Camino Salgado, Sílvia Ruiz-Gaspà, Dolors Costa, Sílvia Beà, Pedro Jares, Elías Campo, Dolors Colomer, Ferran Nadeu

**Affiliations:** 1 Institut d’Investigacions Biomèdiques August Pi i Sunyer (IDIBAPS), Barcelona, Spain; 2 Centro de Investigación Biomédica en Red de Cáncer (CIBERONC), Madrid, Spain; 3 Secció Hematopatologia, Servei Anatomia patològica, Centre Diagnòstic Biomèdic (CDB), Hospital Clínic de Barcelona, Spain; 4 Servei d’Hematologia, Hospital Clínic de Barcelona, Spain; 5 Servei d’Hematologia, Hospital Universitari Mútua Terrassa, Spain; 6 Servei d’Hematología, Hospital Parc Taulí, Sabadell, Spain; 7 Servei d’Hematología, Hospital General Universitari de Vic, Spain; 8 Universitat de Barcelona, Spain

Chronic lymphocytic leukemia (CLL) is biologically and clinically very heterogeneous with a compendium of immunogenetic and genomic factors dictating its course.^1–4^ The mutational status of the immunoglobulin (IG) heavy chain variable region (IGHV) and mutations/deletions of *TP53* are the 2 main molecular markers with prognostic and predictive value, and current guidelines recommend their characterization for a full prognostic evaluation and management of the patients.^2,3^ These 2 biomarkers are usually assessed separately using independent Sanger sequencing and/or next-generation sequencing (NGS) experiments in most diagnostic laboratories,^5–7^ whereas copy number alterations (CNAs) including deletions (del) of 17p, 11q, and 13q and trisomy 12 are mostly analyzed by fluorescence in situ hybridization (FISH). In addition, the study of mutations in *BTK*, *PLCG2*, and *BCL2* is recommended at the time of progression after treatment with BTK and/or BCL2 inhibitors. Although not used for clinical-decision making, mutations in genes such as *SF3B1, NOTCH1*, and the recently described IGLV3-21^R110^ mutation^8^ might also have prognostic/predictive value.^4,9–14^ Efforts have been already made to validate capture-based NGS panels that could integrate the analyses of the main gene mutations and CNAs found in lymphoid malignancies, covering multiple diseases in a single assay.^15,16^ Nonetheless, these panels are large, which might increase sequencing costs when aiming for a high coverage to characterize samples with low tumor cell content and/or subclonal mutations. In addition, their robustness to characterize CNAs and gene mutations of prognostic/predictive value in CLL has been demonstrated in relatively small cohorts of CLL patients.^15,16^ Although these panels might define the clonality of a given B-cell neoplasm by the identification of its IG gene rearrangement, their value to characterize full-length IGHV gene somatic hypermutation (SHM) status has not been proven,^15,16^ probably due to sequence similarities between IGHV genes and their hypermutated nature.^17^ To overcome these limitations, a recent study has suggested to characterize the sequence downstream of the rearranged IGHJ gene as a surrogate of IGHV gene SHM status.^17^ Although conceptually of interest, this approach provided a 88.4% concordance for IGHV gene SHM status compared with conventional Sanger sequencing,^17^ which might be insufficient for routine use in diagnostic units. Here, we present the development and validation of a capture-based NGS panel specifically designed for the molecular characterization of CLL. This new NGS panel, named all-CLL, coupled with a robust bioinformatic pipeline, allows the determination of full-sequence IGH V(D)J gene rearrangements, CNAs, and clonal and subclonal gene mutations, including IGLV3-21^R110^.

The all-CLL panel was customized from a predesigned SOPHiA GENETICS capture-based NGS panel to cover 16 CLL driver genes [*TP53*, *NOTCH1*, *SF3B1*, *ATM*, *BIRC3*, *EGR2*, *FBXW7*, *NFKBIE*, *POT1*, *XPO1*, *KRAS*, *MYD88*, *CXCR4*, *BTK*, *PLCG2*, and *BCL2*], 4 CNAs [del(17p), del(11q), del(13q), and trisomy 12], full-length IGH V(D)J gene rearrangements (from FR1 to FR4) and IGHV gene SHM, and full-length IGLV3-21 gene rearrangements allowing the determination of the R110 mutation (region of interest: 74 Kb; Figure 1A; Supplemental Digital Content; Suppl. Tables S1 and S2). Libraries were performed following manufacturer’s recommendations using as input 200 ng of fresh/frozen genomic DNA and were sequenced on a MiSeq instrument (2 × 300 bp, Illumina, mean coverage ≈1500×; Supplemental Digital Content; Suppl. Table S3). The DDM Platform (SOPHiA GENETICS) was used for variant calling and CNAs analyses. A new version of IgCaller^18^ (v1.3) suitable for capture-based NGS data was developed and used to reconstruct full-sequence IG gene rearrangements, which were subsequently annotated using IMGT/V-QUEST^19^ and ARResT/AssignSubsets^20^ following European Research Initiative on CLL (ERIC) guidelines^5^ (Supplemental Digital Content). To test the all-CLL panel, we initially used a retrospective cohort of 25 purified CLL samples fully-characterized in our previous (immuno)genomic studies.^9–11,14^ This cohort was selected to have a full representation of the main genomic and immunogenetic CLL drivers. We subsequently applied the all-CLL panel in a prospective cohort of 87 patients, in which the tumor cell content of the samples ranged from 19% to 99%. IGHV gene rearrangements and SHM status were analyzed in parallel by Sanger sequencing following ERIC guidelines,^5^ and FISH was performed to determine the status of 17p13.1/*TP53*, 11q22.3/*ATM*, 13q14.3, and trisomy 12 (Figure 1B; Supplemental Digital Content; Suppl. Table S3). In addition, 22 samples from the retrospective (n = 6) or prospective (n = 16) cohorts were analyzed in 2 independent NGS rounds to assess technical reproducibility. A summary of the performance metrices described below can be found in Suppl. Table S4.

**Figure 1.**
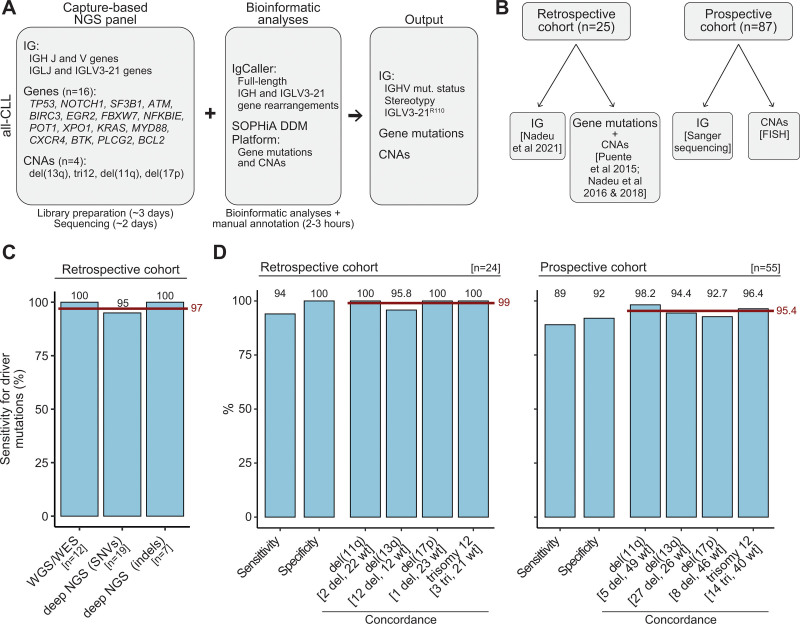
**Design of the all-CLL, validation process, and benchmark of gene mutations and CNAs.** (A) Summary of the all-CLL workflow including the interrogated regions of interest, bioinformatic workflow, and output. Turnaround time of library preparation, sequencing, and bioinformatic analyses are summarized at the bottom. Note that multiple samples can be processed and analyzed in a single experiment (further details can be found in Supplemental Digital Content). (B) Schematic representation of the validation process. (C) Sensitivity of the all-CLL workflow to detect gene mutations according to previously published analyses by WGS/WES^9^ and deep NGS^10,11^; n, number of mutations reported in the previous studies. The maroon crossbar represents the mean value. (D) Sensitivity, specificity, and concordance of CNAs between the all-CLL and gold-standard result in the retrospective (copy number arrays and/or FISH)^9–11^ and prospective cohort (FISH). The maroon crossbar represents the mean concordance value; n, number of cases with CNAs results by all-CLL and gold-standard techniques. Numbers in brackets show the number of samples carrying each alteration (del, deletion; tri, trisomy) or no alterations (wt) by FISH and/or copy number arrays. CNA = copy number alteration; CLL = chronic lymphocytic leukemia; FISH = fluorescence in situ hybridization; indels = short insertions/deletions; NGS = next-generation sequencing; SNV = single-nucleotide variants; WGS/WES = whole-genome/exome sequencing.

Regarding gene mutations in the retrospective cohort, we previously reported 12 mutations in 8 cases by whole-genome/exome sequencing^9^ and 26 mutations in 12 cases by deep NGS (variant allele frequency [VAF] >2%).^11^ No mutations were previously reported in the remaining 5 cases. We identified 37 of 38 (97%) of these mutations using the all-CLL panel (Figure 1C; Suppl. Table S5). The mutation missed (*SF3B1* p.K700E) was previously reported^11^ with a VAF of 5.6% but found at 0.92% of VAF by the all-CLL, which was called but filtered out due to the cutoff of 2% of VAF used during the analysis. The all-CLL panel also identified 19 mutations (7 single-nucleotide variants and 12 indels) in 10 samples that were not previously reported.^11^ Most of these mutations (15/19; 79%) were detected at VAF <6% and appeared to be true positive calls after a careful manual review (Suppl. Figure S1; Suppl. Table S6). Twenty-six mutations were called by the all-CLL assay in the 6 samples analyzed in 2 independent experiments. A reproducibility of 100% was observed for mutations present at a VAF ≥3%, while 3 mutations that were only detected in 1 of the 2 NGS rounds had a VAF <3% (Suppl. Figure S2). In the prospective cohort, at least 1 mutation classified as pathogenic or likely pathogenic was identified in 50 of 87 (57.5%) CLL, including mutations in *BTK*, *PLCG2*, or *BCL2* in samples collected at relapse after treatment with BTK or BCL2 inhibitors, respectively (Supplemental Digital Content; Suppl. Figure S3; Suppl. Table S7). The reanalysis of 16 samples from this prospective cohort in 2 NGS rounds showed a 100% reproducibility for mutations present at a VAF ≥3% (Suppl. Figure S2; Suppl. Table S7).

CNAs were called by the all-CLL panel in 24 of 25 (96%) samples from the retrospective cohort (one sample failed coverage uniformity confidence threshold for CNA analyses). A 99% concordance was observed for the 4 CNAs studied between previously reported results by copy number arrays and/or FISH^9,11^ and the all-CLL panel. The only discrepancy was a del(13q) found in 13% of the cells by FISH in 1 sample, which was missed by the all-CLL panel and copy number arrays (Figure 1D; Suppl. Tables S8 and S9). Fully concordant results were obtained in the 6 samples analyzed twice by all-CLL (Suppl. Table S8). In the prospective cohort, 83 of 87 samples passed the coverage uniformity confidence threshold. Among them, we identified 71 CNAs including 10 del(17p), 7 del(11q), 37 del(13q), and 17 trisomies 12 by all-CLL. FISH analyses performed in parallel in 55 randomly-selected tumors showed a 95.4% concordance, which was similar among the 4 alterations studied (range, 92.7%–98.2%). In addition, a 97% concordance was found in the reproducibility experiment of 16 samples reanalyzed in 2 independent NGS runs (Figure 1D; Suppl. Figure S3; Suppl. Table S10). Combining both cohorts, 12 CLL presented complex karyotype. No differences in the concordance of the CNA calls were observed between CLL with or without complex karyotype (Suppl. Tables S8-S10).

We next assessed the value of the all-CLL workflow to characterize IG gene rearrangements. We detected a full-length, productive IGH V(D)J gene rearrangement in all 25 CLL from the retrospective cohort (Suppl. Table S11). The results were concordant with previously published results^14^ regarding the rearranged IGH V(D)J genes in all tumors and in all but one in terms of CDR3 amino acid sequence (1 amino acid difference in the discordant sample). All tumors were equally classified according to the major stereotyped subsets (1 CLL#1, 4 CLL#2, 1 CLL#8, and 19 unassigned) and as mutated (n = 10) or unmutated (n = 15) IGHV using the 98% cutoff (Figure 2A). In addition, the same percentage of IGHV identity compared with the germ line sequence was determined in virtually all samples, including those with a borderline IGHV identity (97%–97.99%) (Figure 2B; Suppl. Figure S4). This retrospective cohort was intentionally biased toward IGLV3-21-expressing CLL to determine the robustness of the all-CLL panel to define IGLV3-21^R110^ status. Among the 25 CLL studied, 13 tumors expressed the IGLV3-21 gene, either the IGLV3-21*04 (n = 11) or IGLV3-21*02 (n = 2) allele. Among the 11 CLL expressing the IGLV3-21*04, ten carried the R110 mutation.^14^ A concordant result was obtained using the all-CLL approach in all samples (Figure 2C; Suppl. Table S12). In the prospective cohort, a full-length, productive IGH V(D)J gene rearrangement was detected by all-CLL in 83 of 87 (95.4%) samples, 8 belonging to a major stereotyped subset (2 of them CLL#2; Suppl. Table S13). Sanger sequencing performed in parallel in 62 of 87 randomly-selected CLL identified a productive IGH V(D)J gene rearrangement in 57 of 62 (91.9%) samples. When comparing the results of the all-CLL and Sanger sequencing, we found a 100% concordance for IGHV gene SHM status and stereotyped subset assignment, 97.9% concordance regarding the rearranged V(D)J genes identified, and 93.8% in terms of CDR3 amino acid sequence (Figure 2D). In addition, virtually the same IGHV identity compared with the germ line was identified by all-CLL and Sanger sequencing in the 50 full-length IGH rearrangements identified by both techniques, including CLL with borderline IGHV mutations (Figure 2E; Suppl. Figure S5). In this prospective cohort, 5 of 87 (5.8%) CLL carried the IGLV3-21^R110^. In line with previous studies,^13,14^ these 5 CLL included the 2 cases classified as CLL#2 and all 5 carried a borderline IGHV gene SHM status (96.88%–98.26%; Suppl. Figure S3; Suppl. Tables S13 and S14). Finally, the reproducibility experiments in the 2 cohorts showed a 100% concordance for IGHV gene SHM and IGLV3-21^R110^ status and ≥94% concordance in stereotyped subset assignment (Suppl. Figure S6; Suppl. Tables S11-S14).

**Figure 2.**
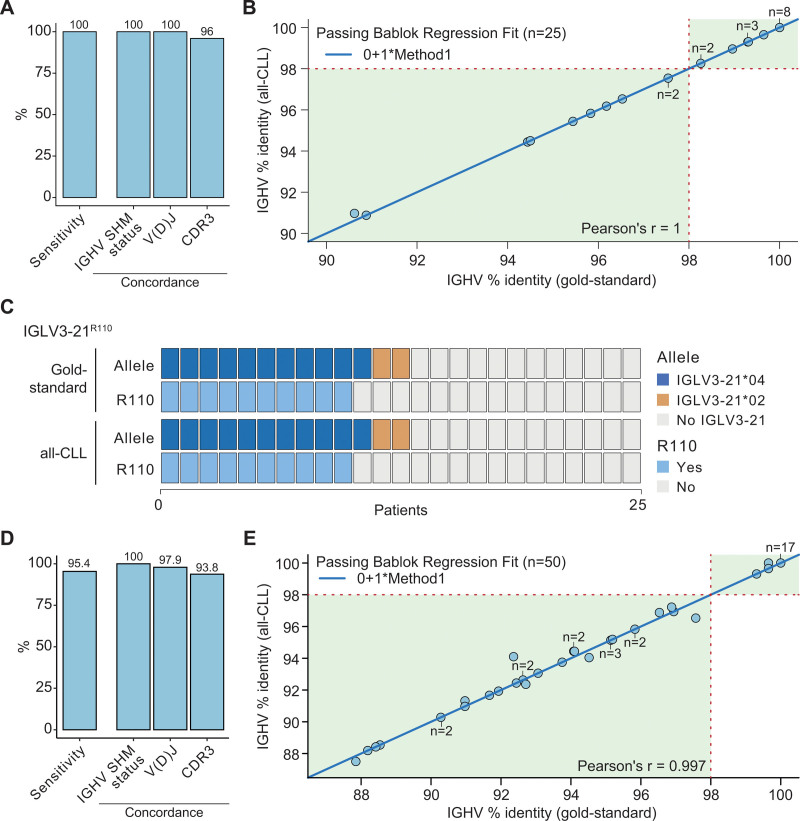
**Benchmark of full-length IGH gene rearrangement, SHM status, and IGLV3-21^R110^.** (A) Sensitivity of the all-CLL workflow to identify a productive IGH gene rearrangement in the retrospective cohort together with the concordance of the IGHV gene SHM status, identified V(D)J genes, and CDR3 amino acid sequence compared with gold-standard results.^14^ (B) Dot plot of the percentage of identity of the rearranged IGHV sequence to the germ line by the all-CLL (y-axis) and gold-standard (x-axis)^14^ in the retrospective cohort. (C) Concordance of the IGLV3-21 gene rearrangement and R110 status identified by the all-CLL compared with gold-standard data.^14^ (D) Sensitivity of the all-CLL to identify a productive IGH gene rearrangement in the prospective cohort together with the concordance of the IGHV gene SHM status, identified V(D)J genes, and CDR3 amino acid sequence compared with Sanger sequencing (gold-standard). (E) Dot plot of the percentage of identity of the rearranged IGHV sequence to the germ line by the all-CLL (y-axis) and gold-standard (x-axis) in the prospective cohort. Full-length IGH gene rearrangements by both approaches were used (n = 50). CLL = chronic lymphocytic leukemia; IGHV = immunoglobulin heavy chain variable region; SHM= somatic hypermutation.

In summary, we have described here the development of the all-CLL, an integrative capture-based NGS solution able to determine in a single experiment the main immunogenetic and genomic markers of prognostic/predictive value in CLL, including full-length IGH V(D)J rearrangements, IGHV gene SHM status, IGLV3-21^R110^, CNAs and driver gene mutations (VAF ≥3%). The all-CLL panel, coupled with the designed bioinformatic workflow, facilitates the molecular characterization of CLL using a single DNA sample as starting material and a single experimental workflow, which reduces costs and turnaround time. The results of this study might thus encourage others to adopt NGS approaches for a more comprehensive (immuno)genomic characterization of CLL, which will contribute to a better understanding of the clinical value of these drivers under distinct treatment options and, ultimately, to advance toward a more personalized management of the patients. Overall, integrative NGS approaches, such as the all-CLL panel presented here, might facilitate the routine molecular characterization of CLL, while providing a complete evaluation of the immunogenetic and genomic drivers of the disease.

## ACKNOWLEDGMENTS

The authors thank the support of Diagnóstica Longwood and SOPHiA GENETICS, and the Hematopathology Collection registered at the Biobank of Hospital Clínic - Fundació de Recerca Clínic Barcelona-Institut d’Investigacions Biomèdiques August Pi i Sunyer (FCRB-IDIBAPS).

## AUTHOR CONTRIBUTIONS

IL-O analyzed and interpreted data, and wrote the article. ML-G analyzed and interpreted data. JC, PM, AM, LM, and A-CS provided samples. SR-G provided logistic support. DC and SB performed fluorescence in situ hybridization (FISH) experiments. PJ supervised next-generation sequencing (NGS) experiments. EC contributed to the design of the study and article preparation. DC designed the study, analyzed and interpreted data, and contributed to article preparation. FN designed the study, analyzed and interpreted data, and wrote the article. All authors reviewed and approved the article.

## DATA AVAILABILITY

Next-generation sequencing (NGS) data have been deposited at the European Genome-phenome Archive (accession number EGAS00001006975).

## DISCLOSURES

EC has been a consultant for Takeda, NanoString, AbbVie, and Illumina; has received honoraria from Janssen, EUSPharma, and Roche for speaking at educational activities and research funding from AstraZeneca and is an inventor on 2 patents filed by the National Institutes of Health, National Cancer Institute: “Methods for selecting and treating lymphoma types,” licensed to NanoString Technologies, and “Evaluation of mantle cell lymphoma and methods related thereof,” not related to this project. DC has received honoraria from AbbVie and AstraZeneca for speaking at educational activities. FN has received honoraria from Janssen, AbbVie, and SOPHiA GENETICS for speaking at educational activities. FN and EC have licensed the use of the protected IgCaller algorithm to Diagnóstica Longwood. All the other authors have no conflicts of interest to disclose.

## SOURCES OF FUNDING

This study was supported by the “la Caixa” Foundation (CLLEvolution—LCF/PR/HR17/52150017 [HR17-00221LCF] and CLLSYSTEMS—LCF/PR/HR22/52420015 [HR22-00172] Health Research 2017 and 2022 Programs, to EC), the Ministry of Science and Innovation (MCIN)/AEI/10.13039/501100011033 and the European Regional Development Fund (FEDER) “Una manera de hacer Europa” (PDC2022-133340-I00 and PID2021-123054OB-I00, to EC and PID2021-123165OB-I00 to DC), European Union NextGenerationEU/Mecanismo para la Recuperación y la Resilencia (MRR)/PRTR and the Instituto de Salud Carlos III (ISCIII) (PMP21/00015 to EC), the Generalitat de Catalunya Suport Grups de Recerca AGAUR (2021-SGR-01172 to EC and 2021-SGR-01294 to DC), and the CERCA program from Generalitat de Catalunya. FN acknowledges research support from the American Association for Cancer Research (2021 AACR-Amgen Fellowship in Clinical/Translational Cancer Research, 21-40-11-NADE), the European Hematology Association (EHA Junior Research Grant 2021, RG-202012-00245), the Lady Tata Memorial Trust (International Award for Research in Leukaemia 2021–2022, LADY_TATA_21_3223). EC is an Academia Researcher of the “Institució Catalana de Recerca i Estudis Avançats” (ICREA) of the Generalitat de Catalunya. This work was partially developed at the Center Esther Koplowitz (CEK, Barcelona, Spain).

## Supplementary Material


