# Supplementary material for: All-CLL: A Capture-based Next-generation Sequencing Panel for the Molecular Characterization of Chronic Lymphocytic Leukemia
Source: Hemasphere. 2023 Sep 21;7(10):e962. doi: 10.1097/HS9.0000000000000962 (PMC10516383; doi:10.1097/HS9.0000000000000962)
Supplement: Supplementary file 1 [file hs9-7-e962-s001.docx]

**Supplementary Data**

**all-CLL: a capture-based next-generation sequencing panel for the molecular characterization of chronic lymphocytic leukemia**

Table of Contents

[Supplementary methods 3](#_Toc138789571)

[Design of the all-CLL panel 3](#_Toc138789572)

[Cohorts 3](#_Toc138789573)

[Library preparation and sequencing 5](#_Toc138789574)

[Gene mutations and CNAs 5](#_Toc138789575)

[Immunoglobulin gene characterization 6](#_Toc138789576)

[Coverage metrics 6](#_Toc138789577)

[Statistical analyses 7](#_Toc138789578)

[List of supplementary tables 8](#_Toc138789579)

[Supplementary figures 9](#_Toc138789580)

[Figure S1. Extended benchmark of gene mutations in the retrospective cohort 9](#_Toc138789581)

[Figure S2. Reproducibility of the variant calling. 10](#_Toc138789582)

[Figure S3. Immunogenetic and genomic landscape of the prospective CLL cohort 11](#_Toc138789583)

[Figure S4. Extended benchmark of the IGHV gene SHM status in the retrospective cohort 12](#_Toc138789584)

[Figure S5. Extended benchmark of the IGHV gene SHM status in the prospective cohort 13](#_Toc138789585)

[Figure S6. Determination of IGHV percentage of identity in two independent NGS rounds 14](#_Toc138789586)

[Supplementary references 15](#_Toc138789587)

# **Supplementary methods**

**Design of the all-CLL panel**

We aimed for a capture-based NGS panel that allowed the determination of the full sequence of IGH gene rearrangements, CNAs, and clonal and subclonal gene mutations, including IGLV3-21^R110^. To that aim, in collaboration with SOPHiA GENETICS, we designed probes to cover:

- FR1 to FR3 region of the IGHV genes and IGHJ genes. Probes targeting the IGHD genes were not designed considering the small size of these genes.
- IGLV3-21 and IGLJ genes.
- Coding and non-coding regions of interest of 16 CLL driver genes (*TP53*, *NOTCH1*, *SF3B1*, *ATM*, *BIRC3*, *EGR2*, *FBXW7*, *NFKBIE*, *POT1*, *XPO1*, *KRAS*, *MYD88*, *CXCR4*, *BTK*, *PLCG2*, and *BCL2*)*.*
- Regions of *ATF1, CDK4, RB1, DLEU1, KLF5, PROZ* and *CUL4A* genes for the analyses of trisomy 12 and del(13q).

The regions of interest are detailed in Supplementary Tables 1 and 2. The panel was named all-CLL (also known as “SOPHiA DDM Community CLL Clonality Solution”).

**Cohorts**

Two independent cohorts of patients with chronic lymphocytic leukemia (CLL) were used for validation purposes. First, a retrospective cohort of 25 CLL with complete genomic^1–3^ and immunogenetic^4^ data available was selected. The sample selection process was based on DNA availability and to fulfill the following requirements:

1. To include CLL samples with clonal and/or subclonal driver gene mutations.
2. To include some CLL samples carrying copy number alterations (CNA) in the four regions studied.
3. To cover the spectrum of IGHV gene somatic hypermutation (SHM) status (90-100% IGHV identity to the germline).
4. To include some CLL belonging clinically relevant stereotyped subsets #2 and #8.
5. To include CLL with and without IGLV3-21 gene rearrangements.
6. To include CLL with and without the R110 mutation in the context of an IGLV3-21 rearrangement.

This retrospective cohort allowed us to benchmark the mutation calling with previously published clonal mutations (variant allele frequency, VAF, >12%) identified by whole-genome/exome sequencing^1^ and/or high-coverage target next-generation sequencing (NGS).^2,3^ Subclonal mutations (VAF <12%) were previously analyzed by target NGS.^2,3^ The samples selected were previously analyzed using standard fluorescence in situ hybridization (FISH) and/or copy-number arrays with complete information for del(17p)/*TP53*, del(11q)/*ATM*, del(13q) and trisomy 12.^1–3^ All 25 samples included also had complete immunoglobulin gene rearrangement information according to a previous study.^4^ The tumor cell content of the samples used ranged from 95% to 99.88% (Supplementary Table 3). Six CLL from this retrospective cohort were analyzed in two independent NGS rounds to assess reproducibility (Supplementary Table 3).

Second, a prospective series of 87 patients with CLL was used to validate the all-CLL panel in the context of a routine molecular diagnostic unit. Therefore, 87 CLL samples sent to the Biomedical Diagnostic Center of the Hospital Clínic of Barcelona for the study of the IGHV gene SHM, *TP53* mutations and/or FISH alterations [del(17p)/*TP53*, del(11q)/*ATM*, del(13q) and trisomy 12] were randomly selected and analyzed using the all-CLL panel. No prior information about tumor purity, IGHV gene SHM, and presence of mutations/CNAs was considered during the selection process. The tumor cell content of the selected samples ranged from 19% to 99% (this information was not available for 32 samples). *TP53* mutations as well as mutations in any of the remaining genes included in the NGS panel were not subjected to orthogonal validation in this prospective cohort. IGH gene rearrangement and SHM status were analyzed in parallel for 62 randomly-selected samples with available material by Sanger sequencing on genomic DNA and/or complementary DNA using leader and consensus constant primers following the European Research Initiative on CLL (ERIC) guidelines.^5^ The whole IGHV region was covered in 52/60 rearrangements found in 57/62 CLL samples analyzed (Supplementary Table 13). Fifty out of the 52 full-sequence rearrangements were also identified by the all-CLL and, therefore, these 50 CLL were used to compare the IGHV percentage of identity detected by Sanger sequencing and NGS. A total of 59 samples with available material were randomly selected and subjected to FISH analyses to determine the status of 17p13.1/*TP53*, 11q22.3/*ATM*, 13q14.3 and trisomy 12. FISH was performed in fixed cells using the XL CLL Probe Kit (Metasystems) performing two hybridizations per case. Hybridization and detection were performed according to the manufacturer's protocols. Two hundred nuclei were examined for each probe. According to our internal controls, the cut-offs used were 11% for *TP53*, *ATM*, and 13q14.3 heterozygous deletions, 4.8% for 13q14.3 homozygous deletions, and 4.1% for trisomy 12. Evaluable results were obtained for 55 samples. Sixteen CLL from the prospective cohort were analyzed in two independent NGS rounds to assess reproducibility (Supplementary Table 3).

The study was approved by the Hospital Clínic of Barcelona Ethics Committee. Informed consent was obtained for all patients.

**Library preparation and sequencing**

Tumor DNA from the retrospective cohort was obtained from the Biobank of Hospital Clínic - Institut d’Investigacions Biomèdiques August Pi i Sunyer (IDIBAPS). DNA was previously obtained from purified fresh or cryopreserved tumor cells. The same DNA samples were used in the previous studies considered as “gold-standard” results.^1,3,4^ Tumor DNA from the samples included in the prospective cohort was obtained from peripheral blood or bone marrow. Library preparation was performed following SOPHiA GENETICS recommendations using 200 ng genomic DNA as input and xGen® Lockdown® Probes from IDT provided by SOPHiA GENETICS. Libraries were sequenced on a MiSeq instrument (2x300 bp, Illumina) aiming at a mean coverage of 1,000x. Turnaround time for library preparation is 3 days plus 2 additional days of sequencing. Note that library preparation and sequencing can be performed in batches of multiple samples.

**Gene mutations and CNAs**

Gene mutations and CNAs were analyzed using the DDM^TM^ Platform from SOPHiA GENETICS using default parameters. The DDM^TM^ Platform uses as input the raw sequencing reads (FASTQ files), aligns them to the GRCh37/hg19 reference genome, and performs the mutation calling and annotation as well as the analysis of CNAs. CNA alterations from the retrospective and prospective cohort were analyzed using the copy number variation (CNV) detection configuration CCLL_A_v3 v11.23 and v12.5-36, respectively. The CNV algorithm performs a double normalization of coverage levels across target regions and across samples, using reference sets of multiple samples in the same analysis run. These reference sets, chosen automatically and individually for each sample, are constructed from other samples that share similar coverage characteristics. Next, a hidden Markov model is used to determine the most probable copy number for each target region. This also allows CNV calls in each target region to be classified as high confidence, medium confidence or undetermined. In addition, samples are classified into low noise, medium noise or rejected based on the residual coverage noise after normalization and CNV calling. CNV results are not provided for rejected samples. The algorithm can detect both germline and somatic CNVs. Since somatic CNVs may affect only a fraction of cells, copy numbers may be fractional. The algorithm accounts for this by using a suitably discretized set of fractional copy numbers. Note that an external panel of normal samples is not used in the analysis.

Gene mutations in the retrospective cohort were analyzed using the variant calling pipeline from the DDM^TM^ Platform (Pipeline ID: ILLXG1S5_CNV11; revision number: v5.5.67). The list of variants reported was first filtered to select potential functional mutations meeting the following criteria: i) variant type (SNP or INDEL), ii) inframe, frameshift, missense, nonsense, no-start, no-stop, splice site or UTR variant (only *NOTCH1* 3’UTR hotspot mutations^1^ considered), iii) variant allele frequency ≥2%, iv) frequency in GnomAD^6^ ≤0.02, and v) frequency in each batch of 8 samples analyzed together in a single run ≤0.3. The latter criterion was revised manually and not followed when it was referred to a hotspot mutation. Only the list of mutations passing these criteria were used for comparison purposes in the retrospective cohort since only somatic mutations were reported in our previous study using whole-genome/exome sequencing^1^ and a similar workflow was used in our previous target NGS analyses.^3^ In the prospective cohort, the pathogenicity of the mutations passing the previous criteria was determined using Franklin (https://franklin.genoox.com), VarSome,^7^ ClinVar,^8^ and Seshat^9^ databases and contrasted with a literature search. The standards for the classification of pathogenicity of somatic variants in cancer^10^ were followed.

A complete analysis of gene mutations and CNA from FASTQ files to final annotations takes 2-3 hours per sample. Note that the bioinformatic steps of the analysis, which takes 1-2 hours, can be executed in parallel for all samples within a batch. The downstream manual review usually takes 15-60 minutes per sample.

**Immunoglobulin gene characterization**

Raw sequencing reads (FASTQ files) were aligned to the GRCh38.p13 (GCA_000001405.28) reference genome using BWA-MEM algorithm (v0.7.17)^11^ using the following parameters: -B 2 -O 6,6 -L 3,3. SAM files were converted to BAM files and BAM files sorted and indexed using samtools (v1.9, with default parameters).^12^ PCR and/or optical duplicates were marked using picard MarkDuplicates (v2.24.0, with default parameters; https://broadinstitute.github.io/picard). IgCaller (v1.3)^13^ was run using the following optional parameters: -seq capture -mntoncoPass 20 -vaf 0.5,0.1 -d 1 -s 10. The online IMGT/V-QUEST tool was used to annotate the sequences of the IGH and IGL gene rearrangements obtained by IgCaller using default parameters with the options “Search for insertions and deletions in V-REGION” and “Clinical application: search for CLL subsets #2 and #8” selected.^14,15^ The online ARResT/AssignSubsets tool was used to further study stereotypy.^16^ A complete IG analysis from FASTQ files to the final annotation takes ~20 minutes per sample. Note that the bioinformatic steps of the analysis, which takes 10-15 minutes per sample, can be parallelized.

**Coverage metrics**

Coverage metrics were obtained using the CollectTargetedPcrMetrics function from picard (v2.24.0, https://broadinstitute.github.io/picard) with the following parameters
-MINIMUM_MAPPING_QUALITY 0 -MINIMUM_BASE_QUALITY 10.

**Statistical analyses**

Comparison of the IGHV percentage of identity to the germ line between the all-CLL panel and gold-standard data was performed using the Passing-Bablok regression (mcr R package v1.2.2), Pearson correlation coefficient (stats R package v4.1.3), and Bland-Altman plot using the bland.altman.stats from BlandAltmanLeh R package (v0.3.1). All analyses were performed in R (v4.1.3).

# **List of supplementary tables**

*Supplementary Tables are placed in the Supplementary Tables Excel file.*

Table S1. Summary of the all-CLL panel

Table S2. Captured regions

Table S3. Patients, samples and sequencing metrics

Table S4. Summary of performance metrices

Table S5. Gold-standard gene mutations in the retrospective cohort according to WGS/WES and/or NGS

Table S6. Gene mutations called by the all-CLL in the retrospective cohort

Table S7. Gene mutations called by the all-CLL in the prospective cohort

Table S8. Copy number alterations in the retrospective cohort

Table S9. Genome-wide copy number alterations in the retrospective cohort previously identified by SNP arrays

Table S10. Copy number alterations in the prospective cohort

Table S11. Retrospective IGH characterization

Table S12. Retrospective IGLV3-21^R110^ characterization

Table S13. Prospective IGH characterization

Table S14. Prospective IGLV3-21^R110^ characterization

# **Supplementary figures**

**Figure S1. Extended benchmark of gene mutations in the retrospective cohort. A.** Bar plots showing the fraction of mutations called by the all-CLL approach that were reported in our previous studies^1,3^ (single nucleotide variants, SNVs, and short insertions/deletions, indels).
**B-D.** Examples of indels detected by the all-CLL workflow that were missed in previous analyses.


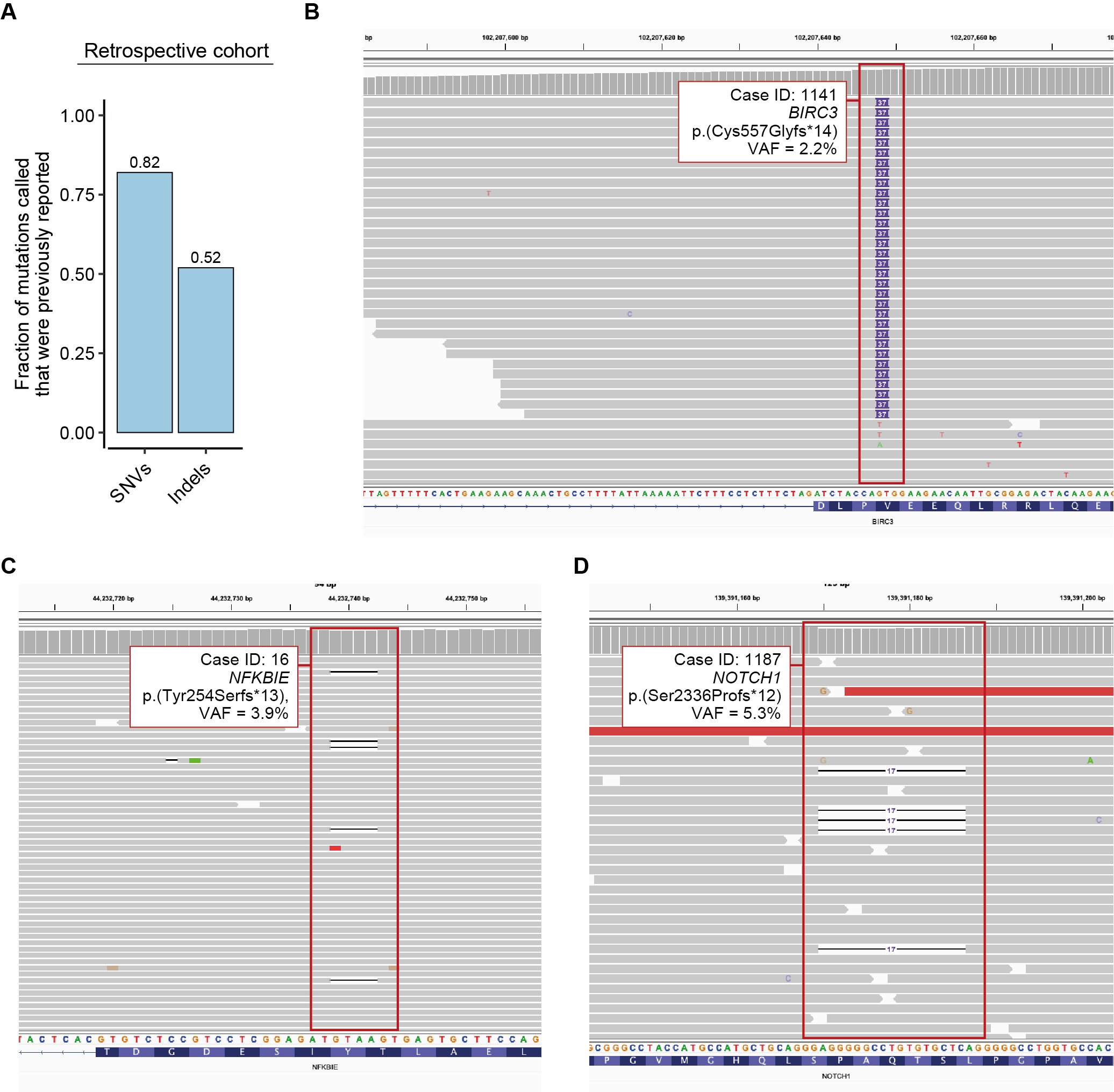


**Figure S2. Reproducibility of the variant calling.** Dot plot showing the correlation and concordance of the variant allele frequency (VAF) of the mutations identified in the 6 samples of the retrospective cohort (**A**) and 16 samples from the prospective cohort (**B**) analyzed in two independent NGS rounds (run 1 and run 2) using the all-CLL panel. Red, dashed line indicates the 2% VAF cutoff used for variant calling.

**
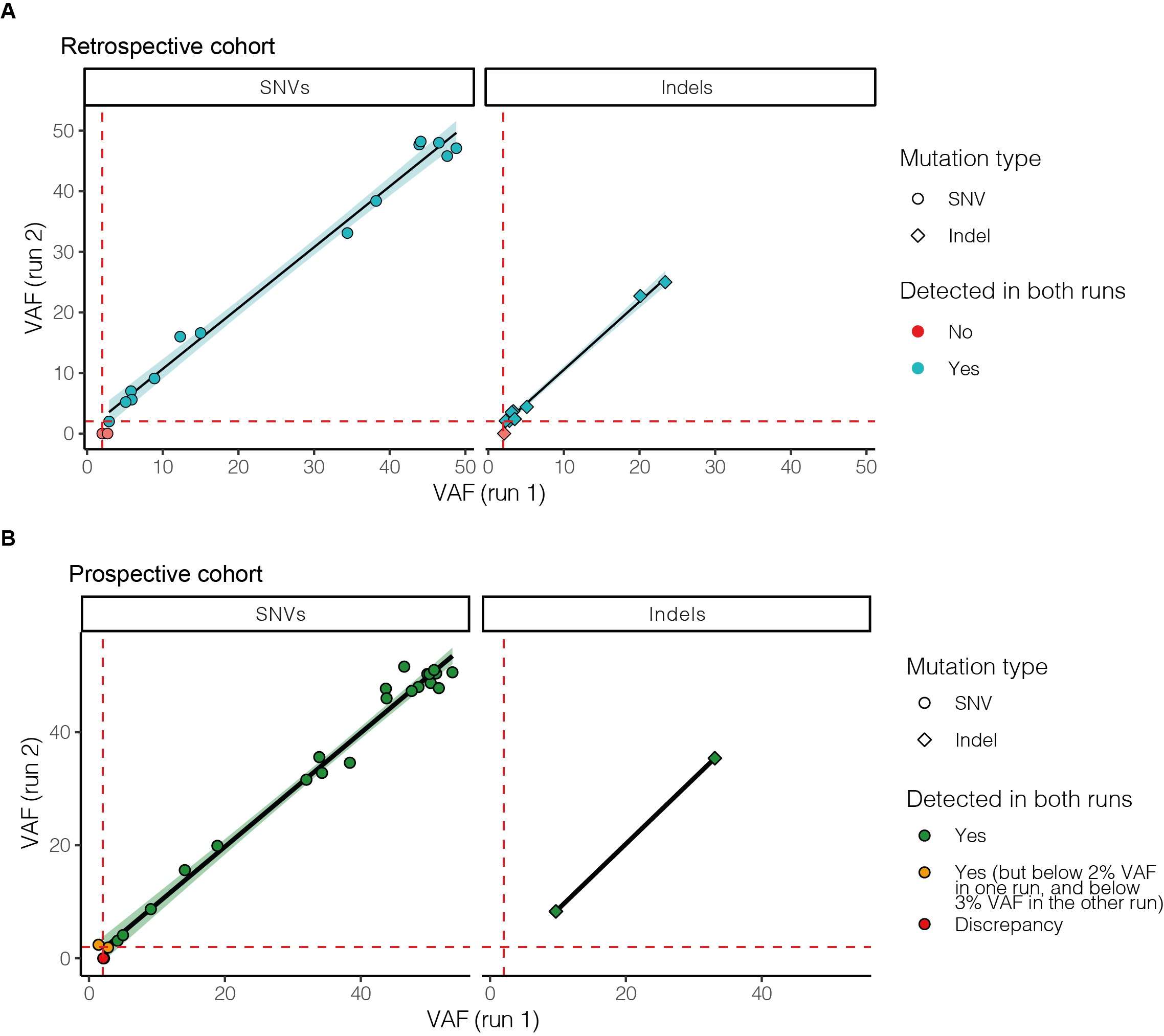
**


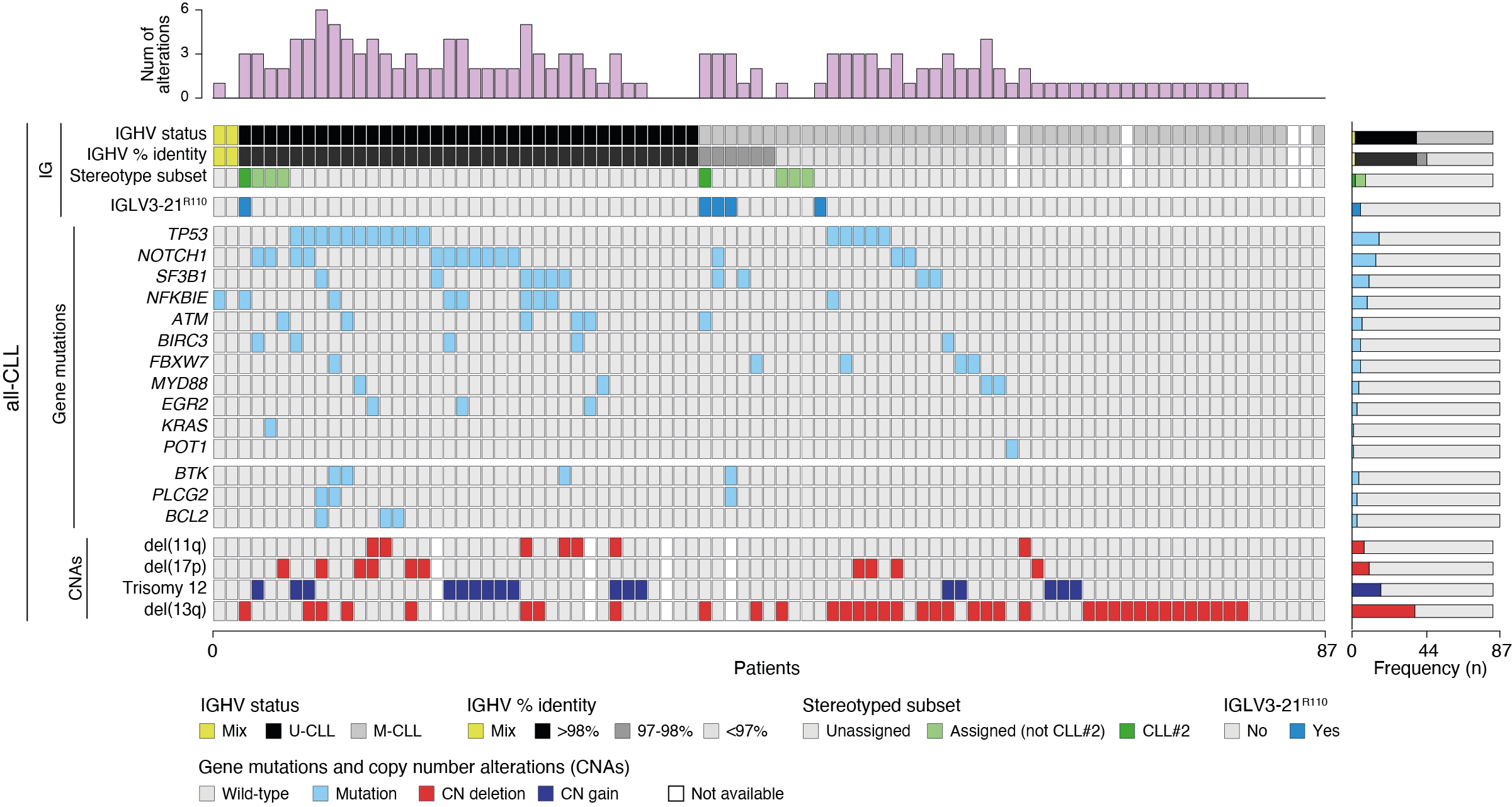
**Figure S3. Immunogenetic and genomic landscape of the prospective CLL cohort.** Oncoprint representation showing the IGHV gene SHM status, IGHV percentage of identity, stereotyped subset, IGLV3-21^R110^, driver gene mutations and CNAs identified in the prospective CLL cohort using the all-CLL panel. Only pathogenic or likely pathogenic mutations were considered as drivers. Mutations found in genes included in the design of the panel to capture copy number alterations (CNAs) were not included in this representation. IGHV status “mix” highlights CLLs with two productive IGHV gene rearrangements, one being mutated (M-CLL) and one unmutated (U-CLL).

**Figure S4. Extended benchmark of the IGHV gene SHM status in the retrospective cohort. A.** Bland-Altman plots of the comparison of the percentage of identity of the rearranged IGHV sequence identified by the all-CLL panel and gold-standard data. Solid, dark blue line represents the mean difference, while dashed, light blue lines correspond to the 95% limits of agreement (average distance ±1.96 standard deviation of the difference). **B.** Bar plots showing the percentage of identity of the rearranged IGHV sequenced identified by the all-CLL and gold-standard data. Red line represents the cut-off (98%) used to categorized CLL as mutated or unmutated IGHV. Dashed red line represents 97% of IGHV identity.


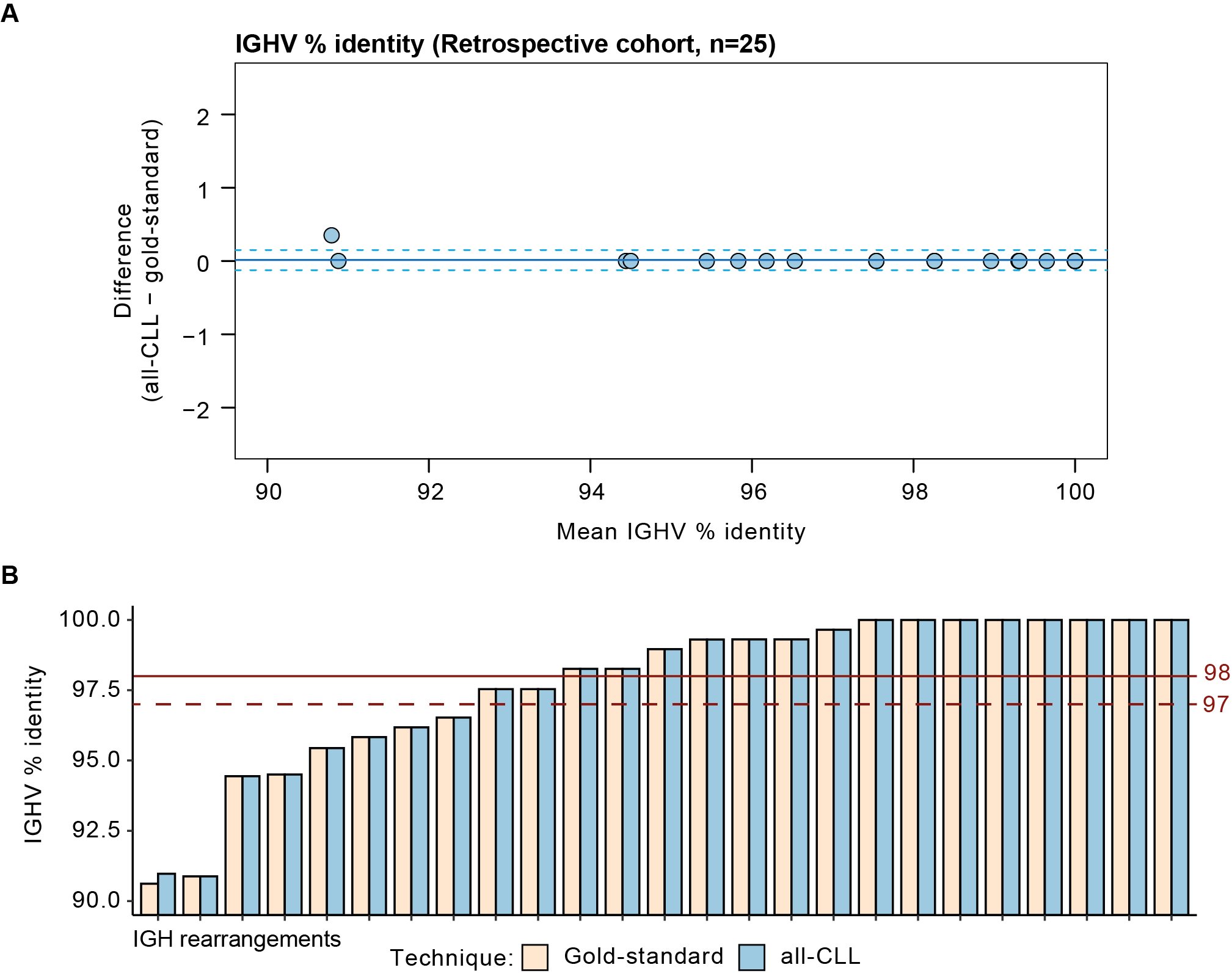


**Figure S5. Extended benchmark of the IGHV gene SHM status in the prospective cohort. A.** Bland-Altman plots of the percentage of identity of the rearranged IGHV sequence identified by the all-CLL and Sanger sequencing. Solid, dark blue line represents the mean difference, while dashed, light blue lines correspond to the 95% limits of agreement (average distance ±1.96 standard deviation of the difference). **B.** Bar plots showing the percentage of identity of the rearranged IGHV sequenced identified by the all-CLL and Sanger sequencing. Red line represents the cut-off (98%) used to categorized CLL as mutated or unmutated IGHV. Dashed red line represents 97% of IGHV identity.


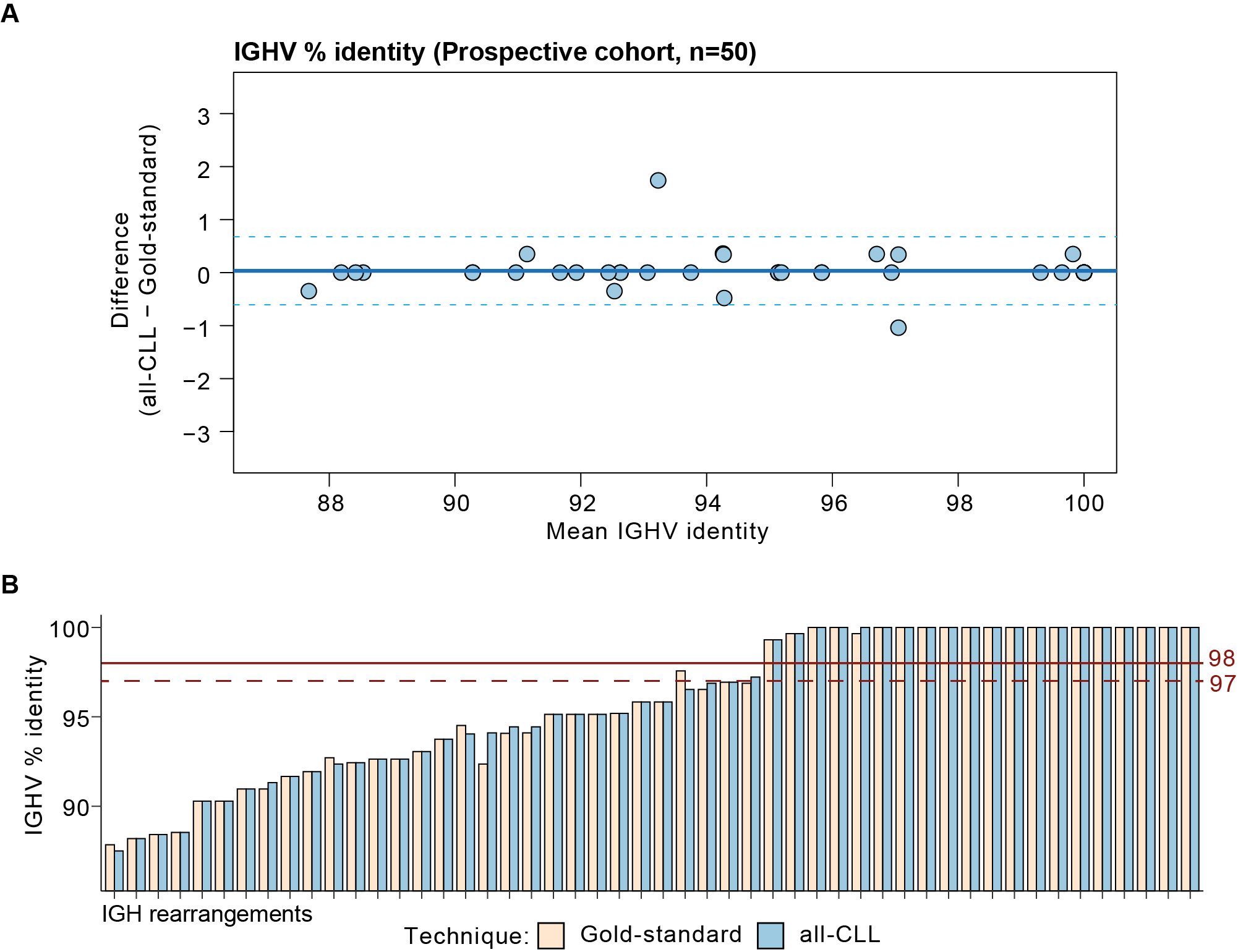


**Figure S6. Determination of IGHV percentage of identity in two independent NGS rounds.** Dot plot of the percentage of identity of the rearranged IGHV sequence to the germ line by the all-CLL in the two independent NGS rounds (Run 1 and Run 2). The sample highlighted as “discrepant case” showing the higher difference in IGHV identity between the two runs was the unique sample in the reproducibility experiments in which slightly different IGHV genes were found rearranged in the two sequencing runs (case 550010658, Supplementary Table 13).


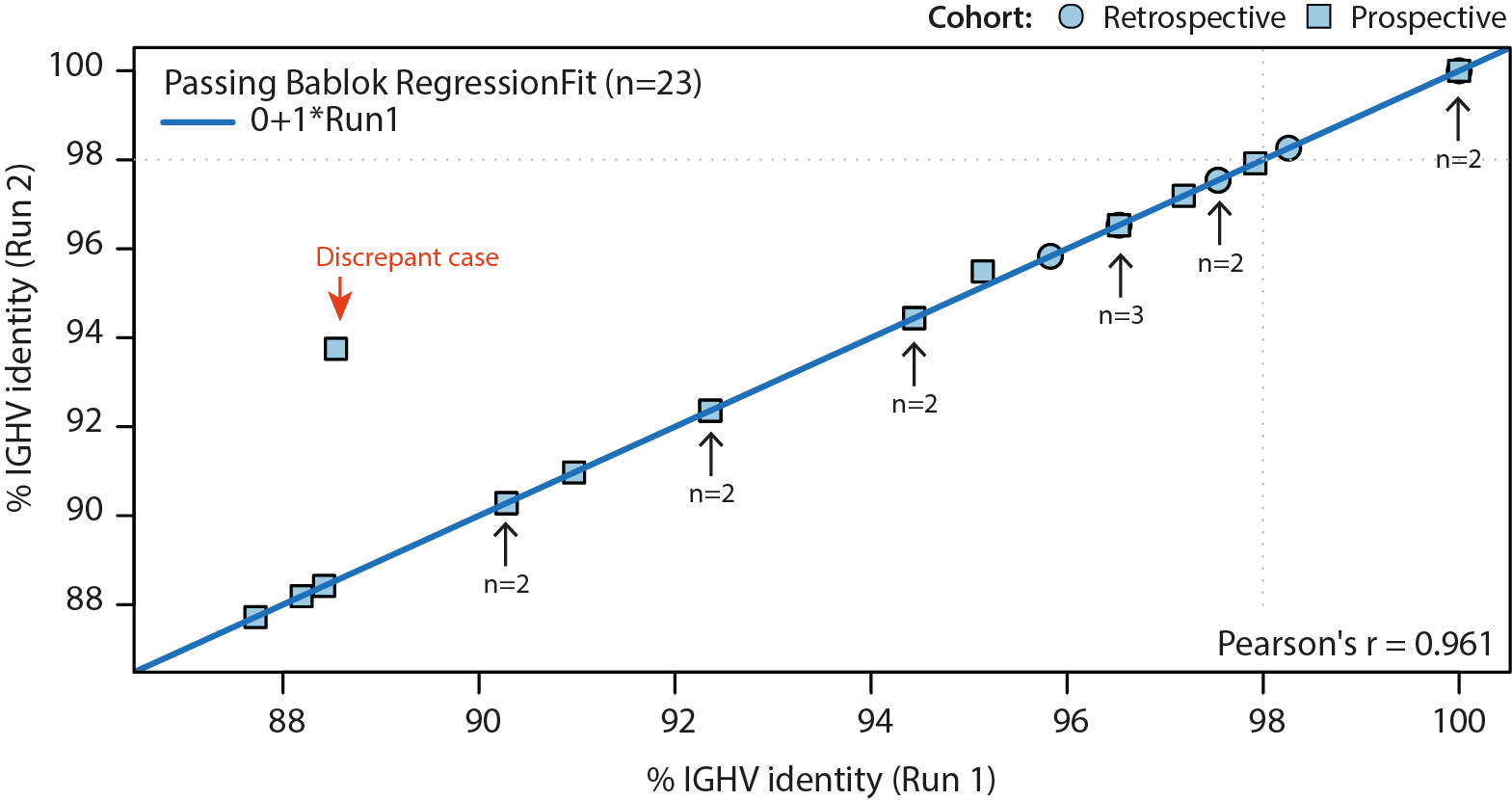


# **Supplementary references**

1. Puente XS, Beà S, Valdés-Mas R, et al. Non-coding recurrent mutations in chronic lymphocytic leukaemia. Nature 2015;526(7574):519–524.

2. Nadeu F, Delgado J, Royo C, et al. Clinical impact of clonal and subclonal TP53, SF3B1, BIRC3, NOTCH1, and ATM mutations in chronic lymphocytic leukemia. Blood 2016;127(17):2122–2130.

3. Nadeu F, Clot G, Delgado J, et al. Clinical impact of the subclonal architecture and mutational complexity in chronic lymphocytic leukemia. Leukemia 2018;32(3):645–653.

4. Nadeu F, Royo R, Clot G, et al. IGLV3-21R110 identifies an aggressive biological subtype of chronic lymphocytic leukemia with intermediate epigenetics. Blood 2021;137(21):2935–2946.

5. Agathangelidis A, Chatzidimitriou A, Chatzikonstantinou T, et al. Immunoglobulin gene sequence analysis in chronic lymphocytic leukemia: the 2022 update of the recommendations by ERIC, the European Research Initiative on CLL. Leukemia 2022;36(8):1961–1968.

6. Karczewski KJ, Francioli LC, Tiao G, et al. The mutational constraint spectrum quantified from variation in 141,456 humans. Nature 2020;581(7809):434–443.

7. Kopanos C, Tsiolkas V, Kouris A, et al. VarSome: the human genomic variant search engine. Bioinformatics 2019;35(11):1978–1980.

8. Landrum MJ, Lee JM, Benson M, et al. ClinVar: improving access to variant interpretations and supporting evidence. Nucleic Acids Res 2018;46(D1):D1062–D1067.

9. Tikkanen T, Leroy B, Fournier JL, Risques RA, Malcikova J, Soussi T. Seshat: A Web service for accurate annotation, validation, and analysis of TP53 variants generated by conventional and next-generation sequencing. Hum Mutat 2018;39(7):925–933.

10. Horak P, Griffith M, Danos AM, et al. Standards for the classification of pathogenicity of somatic variants in cancer (oncogenicity): Joint recommendations of Clinical Genome Resource (ClinGen), Cancer Genomics Consortium (CGC), and Variant Interpretation for Cancer Consortium (VICC). Genet Med 2022;24(9):1991.

11. Li H, Durbin R. Fast and accurate short read alignment with Burrows-Wheeler transform. Bioinformatics 2009;25(14):1754–1760.

12. Li H, Handsaker B, Wysoker A, et al. The Sequence Alignment/Map format and SAMtools. Bioinformatics 2009;25(16):2078–2079.

13. Nadeu F, Mas-de-les-Valls R, Navarro A, et al. IgCaller for reconstructing immunoglobulin gene rearrangements and oncogenic translocations from whole-genome sequencing in lymphoid neoplasms. Nat Commun 2020;11(1):3390.

14. Lefranc M-P, Giudicelli V, Ginestoux C, et al. IMGT, the international ImMunoGeneTics information system. Nucleic Acids Res 2009;37(Database issue):D1006–D1012.

15. Brochet X, Lefranc M-P, Giudicelli V. IMGT/V-QUEST: the highly customized and integrated system for IG and TR standardized V-J and V-D-J sequence analysis. Nucleic Acids Res 2008;36(Web Server):W503–W508.

16. Bystry V, Agathangelidis A, Bikos V, et al. ARResT/AssignSubsets: a novel application for robust subclassification of chronic lymphocytic leukemia based on B cell receptor IG stereotypy. Bioinformatics 2015;31(23):3844–3846.
